# Pericytes: Problems and Promises for CNS Repair

**DOI:** 10.3389/fncel.2019.00546

**Published:** 2019-12-06

**Authors:** Fabio Laredo, Julia Plebanski, Andrea Tedeschi

**Affiliations:** ^1^Department of Neuroscience, Wexner Medical Center, The Ohio State University, Columbus, OH, United States; ^2^Department of Life Sciences, University of Nottingham, Nottingham, United Kingdom; ^3^Discovery Theme on Chronic Brain Injury, The Ohio State University, Columbus, OH, United States

**Keywords:** pericyte, angiogenesis, blood brain barrier (BBB), neurovascular inflammation, fibrotic scar, axon regeneration, neurodegenerative diseases

## Abstract

Microvascular complications are often associated with slow and progressive damage of various organs. Pericytes are multi-functional mural cells of the microcirculation that control blood flow, vascular permeability and homeostasis. Whereas accumulating evidence suggests that these cells are also implicated in a variety of diseases, pericytes represent promising targets that can be manipulated for therapeutic gain. Here, we review the role of pericytes in angiogenesis, blood-brain barrier (BBB) function, neuroinflammation, tissue fibrosis, axon regeneration failure, and neurodegeneration. In addition, we outline strategies altering pericyte behavior to point out problems and promises for axon regeneration and central nervous system (CNS) repair following injury or disease.

## Introduction

Lack of neurological recovery following central nervous system (CNS) trauma and disease is associated with long-term consequences that impair basic body functions including sensation, cognition, locomotion, vision, bladder and bowel movements, all of which negatively impact the independent quality of life. Axon sprouting, regeneration and *de novo* circuit formation can restore function by reconnecting denervated regions of the brain and spinal cord. Revascularization and vascular normalization of these regions is crucial to ensure adequate delivery of oxygen and nutrients needed to sustain the high metabolic demands of performing daily activities essential for maintaining independent living. Under normal physiological conditions, however, axon growth and regeneration are extremely limited in the adult mammalian CNS (He and Jin, 2016; Tran et al., 2018). In fact, the poor regeneration ability of adult CNS neurons (Liu et al., 2010; Tedeschi and Bradke, 2017) and the hostile environment that develops as a result of trauma or disease (Geoffroy and Zheng, 2014; Schwab and Strittmatter, 2014; Chen et al., 2018; Dias et al., 2018) are major obstacles to neurological recovery. Moreover, aberrant reorganization of the microvascular structure and perivascular cell function also interferes with physiological recovery following injury and neurodegenerative disease (Hall et al., 2014; Li et al., 2017; Nortley et al., 2019). In spite of new discoveries and technological applications that allow reprogramming adult mammalian neurons into a growth-competent state and to eliminate extracellular growth inhibitors (O’Donovan et al., 2014; Wang et al., 2015, 2018; Cartoni et al., 2016; Tedeschi et al., 2016, 2019; Fink et al., 2017; Kim et al., 2018; Bray et al., 2019; Kumamaru et al., 2019; Sekine et al., 2019), other extrinsic neuronal factors such as pericytes and pericyte regulation of vascular structure and function have received less attention. Here, we discuss the role of pericytes in health and disease, and then outline strategies altering pericyte behavior that may be considered to leverage significant improvement of neurological outcome in the context of CNS injury and neurodegeneration.

## Pericytes and Angiogenesis

**Figure 1:**
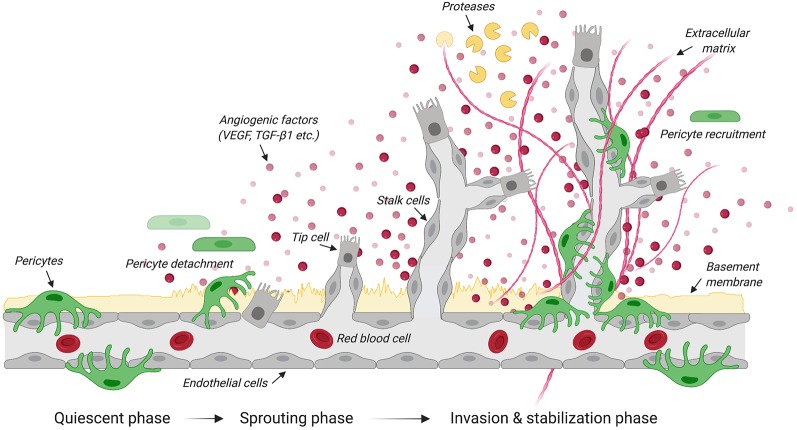
Steps in angiogenesis. Schematic illustrates the quiescent, sprouting and invasion phases that characterize the growth of new blood vessels from the existing vasculature. Upon the release of angiogenic factors, receptor-ligand coupling activates endothelial cells that undergo migration and proliferation. The upregulation of protease expression allows the degradation of the basement membrane and pericyte detachment. Specialized endothelial cells called “tip cells” migrate along the angiogenic gradient where tubule formation is controlled by the Tie-2/angiopoietin axis. Another type of specialized endothelial cells called “stalk cells” proliferates to support the main body of the new vessel. By dissolving the extracellular matrix, proteases contribute to structural remodeling of the tubular network. Pericytes are then recruited to stabilize newly formed blood vessels. Created with biorender.com.

Angiogenesis is the growth of blood vessels from the existing vasculature in both health and disease (Figure 1; Carmeliet, 2003). In all metabolically active tissues, blood capillaries are necessary for the diffusion of nutrients and metabolites as well as the elimination of waste materials. The communication between endothelial cells and pericytes is key for new blood vessel formation, maturation and maintenance. Previously known as “Rouget cells” after the French physiologist Charles Marie Benjamin Rouget who discovered them, pericytes were first named by Zimmermann in 1923. Pericytes directly communicate with endothelial cells *via* physical contact and paracrine signaling (Bergers and Song, 2005). While gap junctions provide direct connections between pericytes and endothelial cells, adhesion plaques and peg-and-socket contacts (e.g., membrane invaginations extending from either cells) allow pericytes to transfer contractile forces to the endothelium (Allt and Lawrenson, 2001; Bergers and Song, 2005). Given that a single pericyte can establish contacts with multiple endothelial cells, pericyte coverage can vary based on blood vessel function and location (Hirschi et al., 1999; Armulik et al., 2005, 2011). During development, transforming growth factor β 1 (TGF-β1) promotes differentiation of pericyte progenitor cells expressing platelet derived growth factor receptor beta (PDGFRβ). These cells are then attracted in the capillary plexus by endothelial cells expressing platelet derived growth factor subunit B (PDGF-B; Hellstrom et al., 1999). PDGF-B is secreted as PDGF-BB homodimers (Andrae et al., 2008). Upon binding to PDGFRβ, PDGF-BB leads to receptor dimerization and phosphorylation thereby activating a number of downstream signaling pathways including phosphoinositide 3-kinase (PI3K), RasGAP and ERK that control cell proliferation and migration (Tallquist and Kazlauskas, 2004). Blood vessels are maintained in a stable equilibrium by Notch-dependent contact inhibition between pericytes and endothelial cells that prevents the latter from proliferating and migrating (Hellstrom et al., 1999; Taylor et al., 2002; Leslie et al., 2007). The importance of PDGF-B in mediating pericyte recruitment to angiogenic vessels is highlighted by experimental data showing that capillaries in PDGF-B null mice show pericyte loss that leads to renal, cardiovascular and hematological abnormalities as well as perinatal death (Leveen et al., 1994). Blocking the recruitment of mural cells (e.g., vascular smooth muscle cells and pericytes) to developing retinal vessels in neonatal mice through daily intraperitoneal injection of a monoclonal antibody directed against PDGFRβ causes detrimental changes in vascular architecture of the retina (Uemura et al., 2002). Of interest, intraocular administration of recombinant angiopoietin-1, a secreted protein produced by pericytes, in these mice partly restores the network of growing blood vessels and vascular function in the absence of mural cells (Uemura et al., 2002). Both angiopoietin-1 and its tyrosine kinase receptor Tie2 expressed in endothelial cells are key to angiogenesis, not only during development, but also in response to injury (Davis et al., 1996; Suri et al., 1996; Jeansson et al., 2011). Once formed, the capillary plexus undergoes extensive structural remodeling through processes of angiogenesis, structural adaptation, sprouting and pruning (Gerhardt and Betsholtz, 2003; Bergers and Song, 2005).

Originally described as an endothelial cell-specific mitogen, the secreted polypeptide vascular endothelial growth factor (VEGF) initiates angiogenesis (Leung et al., 1989). VEGF stimulates endothelial cells to degrade the vascular basement membrane (Daubon et al., 2016), allowing these cells to then invade the extracellular space and migrate along a VEGF gradient (Bergers and Song, 2005). Under hypoxic conditions, pericytes produce VEGF (Shweiki et al., 1992; Darland et al., 2003), detach from the vasculature prior to the migration of endothelial cells and secrete VEGF to create a gradient to guide endothelial cells (Krueger and Bechmann, 2010; Wong et al., 2015). VEGF null mouse embryos display abnormal blood vessel development resulting in embryonic death at mid-gestation (Carmeliet et al., 1996). A similar phenotype is also observed in embryos lacking the VEGF receptor tyrosine kinase Flk-1 (Shalaby et al., 1995), further confirming the importance of VEGF signaling during early vasculogenesis.

**Figure 2:**
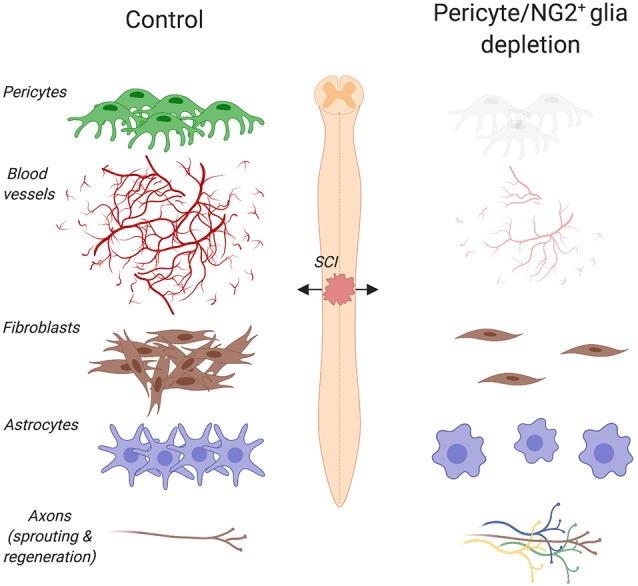
Pericyte/NG2^+^ glia depletion and its effects on the pathophysiology of spinal cord injury (SCI). Ablation of pericytes and NG2^+^ glia impairs angiogenesis, dampens astrogliosis and completely abolishes fibrotic scar formation at the lesion site. Under such pathophysiological conditions, a greater axon regrowth into the lesion is observed. Created with biorender.com.

Pericytes are among the first cells to invade the lesion site in experimental models of brain and spinal cord injury (SCI; Göritz et al., 2011; Birbrair et al., 2014; Dias et al., 2018; Hesp et al., 2018), presumably contributing to the remodeling of the vasculature after trauma. PDGFRβ is one of the major keys to angiogenesis and pericyte function after injury. In fact, endothelial cells secrete PDGF-BB that binds the PDGFRβ expressed on pericytes, stimulating pericyte proliferation and migration to the lesion site (Gaceb et al., 2018a). Human brain pericytes treated with PDGF-BB increase the production of neuroprotective and angiogenic growth factors, including brain-derived neurotrophic factor (BDNF), basic fibroblast growth factor (FGFb) and VEGF (Gaceb et al., 2018b). In contrast, stimulation with lipopolysaccharide (LPS), which is the major component of the outer membrane of Gram-negative bacteria, promotes the secretion of high amounts of proinflammatory cytokines (Gaceb et al., 2018b). Thus, environmental changes profoundly affect pericyte’s functions. Five days after a controlled cortical impact, a well-characterized experimental model that reproduces pathophysiological changes observed in human traumatic brain injury, the proliferation marker Ki67 increases together with PDGFRβ at the lesion core (Zehendner et al., 2015). Similarly, an increase in PDGFRβ/CD13/Ki67 positive cells is observed at the lesion site 5 days after transient focal cerebral ischemia in mice (Fernández-Klett et al., 2013). Other than pericytes, myeloid cells (e.g., monocytes, macrophages, basophils, neutrophils and eosinophils), dendritic cells, epithelial cells of the kidney, small intestine and respiratory tract also express CD13. Increased pericyte density is also observed in glioblastomas and fibrosarcomas, where blood vessels exhibit abnormalities like poor organization, serpentine trajectories and branching irregularities (Nagy et al., 2009). Blocking PDGFRβ signaling using a receptor tyrosine kinase inhibitor in a mouse model of pancreatic islet carcinogenesis causes pericyte detachment and regression of tumor blood vessels (Bergers et al., 2003). Following a cervical contusion injury of the mouse spinal cord, elimination of proliferating pericytes completely abolishes angiogenesis at the site of injury (Hesp et al., 2018; Figure 2), thereby providing further evidence of pericytes being involved in angiogenesis not only in health but also injury and disease.

The proliferation of pericytes after injury and their role in angiogenesis may help axons regenerating across the lesion site by providing vascular bridging and growth permissive substrates for axon growth. Under physiological conditions, however, pericytes do not support axon growth (Göritz et al., 2011). Nonetheless, experimental evidence has shown that upon reactivation of axon growth programs, several classes of regeneration-competent axons including dorsal column sensory, rubrospinal, nociceptive and propriospinal axons preferentially associate with NG2 and CD13 (e.g., common markers for pericytes) positive cells. This provides a tangible prospect of using pericyte bridges to encourage axon regeneration through the lesion site (Jones et al., 2003; McTigue et al., 2006; Anderson et al., 2018). Implementation of chronic multiphoton imaging strategies of the mouse spinal cord allows changes in vascular and axonal networks to be monitored over the course of days and months after injury (Farrar et al., 2012; Fenrich et al., 2012; Tedeschi et al., 2016). Repetitive imaging of the injured spinal cord shows a transient increase in blood vessel density at the site of injury, where interaction with growing axons can be observed within the first 2 weeks after trauma (Dray et al., 2009). In mice, axons growing on blood vessels are eight times longer on average compared to those not following vessels (Dray et al., 2009). Unlike mammals, lower vertebrate species such as zebrafish retain the ability to regenerate injured tissues including the brain, retina and spinal cord (Gemberling et al., 2013; Rasmussen and Sagasti, 2017). In zebrafish larvae, peripheral macrophages promote successful axon regeneration and spinal cord repair by dynamically controlling the expression of the proinflammatory cytokines interleukin-1 β (IL-1β) and tumor necrosis factor-α (TNF-α; Tsarouchas et al., 2018). In mammals, macrophage-induced blood vessels are key for nerve regeneration in the peripheral nervous system (Cattin et al., 2015).

There has long been an interest in exploiting neurovascular interaction to guide axon growth and regeneration. Under appropriate conditions, certain types of pericytes may indeed serve as a primer to promote revascularization at the lesion site, as well as improve axon viability and regeneration.

## Pericytes, the Blood-Brain Barrier, and Neurovascular Inflammation

The ability of pericytes to adapt to their local environment, either by releasing VEGF in response to hypoxia or by secreting growth factors in response to PDGF-BB, is evidence of their versatility and all-encompassing responsibilities (Rustenhoven et al., 2017; Gaceb et al., 2018b). The adaptability of these cells also includes responding to and mediating inflammatory signals (Rustenhoven et al., 2017).

Pericytes release low levels of chemokines spontaneously (Kovac et al., 2011; Göritz et al., 2011). After being treated by inflammatory signals such as LPS or TNF-α, pericytes significantly increase their production of chemokines and cytokines, such as the chemokine (C-X-C motif) ligand 1 (CXCL1) and interleukin-6 (IL-6), as well as increasing the number of receptors for inflammatory signals, such as Toll-Like Receptor 4 (TLR4; Stark et al., 2013; Guijarro-Muñoz et al., 2014). Pericyte stimulation with LPS causes translocation of the nuclear factor kappa-B (NF-kB) protein complex, a prototypical pro-inflammatory signaling pathway (Lawrence, 2009), into the nucleus with consequent activation of its downstream effectors (Guijarro-Muñoz et al., 2014). The upregulation of inflammatory receptors and activation of key pro-inflammatory pathways indicate that pericytes not only respond to inflammation but also are critical modulators that can directly affect inflammation. However, pro-inflammatory modulation is not always in the best interests of the surrounding environment: pericytes conditioned by TNF-α activate neutrophils and monocytes, also engaging with and activating microglia (Proebstl et al., 2012; Stark et al., 2013). All these are actions that have potential neurodegenerative effects.

In addition to secreting and responding to inflammatory signals, pericytes also have the ability to directly interact with inflammatory cells such as monocytes and leukocytes (Rustenhoven et al., 2017). When pericytes are placed in an inflammatory environment *in vivo*, they upregulate expression of adhesion molecules, such as intercellular adhesion molecule-1 (ICAM-1), that allow binding and transmigration of leukocytes across the blood-brain barrier (BBB; Yang et al., 2005; Persidsky et al., 2016). Chemokines such as C-C motif chemokine (CCL)2 and CCL3 released from pericytes actively attract and recruit monocytes and T cells, thus encouraging the flow of inflammatory cells to the area (Rustenhoven et al., 2017). The cells then bind to pericytes through upregulated adhesion molecules and are transferred across the BBB (Rustenhoven et al., 2017). A study researching the function of brain pericytes in human immunodeficiency virus (HIV) positive individuals, a disease characterized by chronic neuroinflammation, has discovered that these individuals show decreased pericyte number and expression of pericyte markers PDGFRβ and CD13 (Persidsky et al., 2016). Such a decrease in pericyte number coincides with a typical increase in activated microglia when compared to healthy controls (Persidsky et al., 2016), identifying pericytes as a significant component of chronic neuroinflammation in HIV-positive individuals. These results suggest that pericytes may contribute to control neuroinflammation; perhaps through indirect relationships with microglia, as the decrease in pericytes is followed by an increase in activated microglia (Persidsky et al., 2016). Further evidence of pericytes being able to modulate neuroinflammation is described in a study that researched the effects of human *APOE4*, a major genetic risk factor for Alzheimer’s Disease (AD), and a lack of murine *Apoe* on proinflammatory cyclophilin A expression (Bell et al., 2012). Both animal models display an inability to control cyclophilin A expression in pericytes, resulting in an increased activation of NF-kB and matrix metalloproteinase 9, which in turn leads to BBB breakdown (Bell et al., 2012). BBB dysfunction is a treatable insult, as antagonists for all three components of the cyclophilin A/NF-kB/matrix metalloproteinase 9 pathway in pericytes reverse the leaky BBB phenotype (Bell et al., 2012), identifying pericytes as a promising therapeutic target.

The healthy CNS has restricted leukocyte access under homeostatic conditions. During an inflammatory response, however, leukocytes move more freely across the BBB or blood spinal cord barrier (Rustenhoven et al., 2017). The ability of leukocytes to move more freely may rest with how pericytes react to the inflammatory response. The BBB is regulated by the neurovascular unit comprised of vascular cells (e.g., endothelial, pericytes and vascular smooth muscle cells), glial cells (e.g., microglia, astrocytes, and oligodendroglia) and neurons (Zlokovic, 2011). Although pericytes’ involvement in the neurovascular unit is traditionally thought of as exclusive to capillaries, they are also present on post-capillary venules (Attwell et al., 2016). In particular, pericytes and endothelial cells form tight junctions that allow specific movement of molecules across this barrier, with pericytes playing a vital role in the maintenance of these junctions (Daneman et al., 2010). Transmigration of neutrophils from the vascular lumen into the surrounding tissues is not only an important response for the host’s defense but also a major cause of inflammation. Multidimensional imaging using intravital microscopy shows neutrophils using pericytes for crawling purposes on the endothelium, thereby providing convincing evidence of direct interactions between these two cell types *in vivo* (Proebstl et al., 2012). As mentioned above, pericytes stimulation with TNF-α significantly upregulates ICAM-1. When a blocking antibody directed against ICAM-1 is added *in vivo*, neutrophil crawling on pericytes significantly decreases, suggesting that adhesion between the neutrophils and pericytes is essential in neutrophil migration (Proebstl et al., 2012). Pericytes do not solely act as a “railway” for migrating neutrophils. TNF-α stimulation for 24 h increases gaps between pericytes and endothelial cells, therefore allowing more neutrophils to breach the venule wall (Proebstl et al., 2012; Stark et al., 2013). Furthermore, neutrophils that interact with pericytes move more quickly and directly to their final destination (Proebstl et al., 2012; Stark et al., 2013), demonstrating that pericytes are not just passive cellular players in neuroinflammation, but actively instruct inflammatory cells.

The integrity of the BBB is critical for maintaining a homeostatic environment in the CNS to allow normal functionality (Zlokovic, 2011). Of the cells comprising the neurovascular unit, recent studies have emphasized the importance of pericytes in developing and maintaining its structure (Armulik et al., 2010; Daneman et al., 2010; Villaseñor et al., 2017). Astrocytes’ major contribution to BBB formation is well established (Janzer and Raff, 1987). However, BBB tight junction molecules such as occludin, claudin 5 and zonula occludens-1 (ZO-1) are expressed as early as embryonic day 12 in mice, as is the BBB influx transporter Glut-1 (Daneman et al., 2010). Given that the majority of astrocytes are produced after birth, these results challenge the concept that astrocytes are the only cellular source responsible for inducing endothelial cells to form the tight junction’s characteristic of the BBB. Research into the effects of pericyte deficiency, often using transgenic PDGFβ^ret/ret^ mice (c.a. 80% reduction in pericyte coverage) where PDGF-B binding to heparan sulfate proteoglycans is disrupted and PDGFRβ^F7/F7^ mice which have approximately 40% of the pericytes coverage of control littermates, have elucidated the impact that loss of pericytes has on the BBB (Lindblom et al., 2003; Armulik et al., 2010; Daneman et al., 2010; Nikolakopoulou et al., 2017). PDGFRβ null mice are embryonically lethal as these mice completely lack CNS pericytes (Leveen et al., 1994). In contrast to null mice, mice that are PDGFRβ deficient still produce CNS pericytes but at a much lesser volume than healthy controls. Interestingly, PDGFRβ^F7/F7^ mice, an experimental model with impaired downstream PDGFRβ signaling, display a reduction in microcirculation, BBB breakdown and extravascular increase in IgG, fibrinogen and fibrin deposits in the CNS (Bell et al., 2010; Nikolakopoulou et al., 2017; Montagne et al., 2018). These studies also highlight a negative correlation between the number of pericytes and BBB breakdown measurements, all evidence of BBB damage upon pericyte depletion. Increased vascular permeability in PDGFRβ deficient mice is partly due to the increase in transcytosis across the BBB (Rustenhoven et al., 2017). This phenomenon may not be surprising, especially considering the importance of pericytes in forming tight junctions with endothelial cells. Yet, the PDGFβ^ret/ret^ mice express normal levels of tight junction molecules (Villaseñor et al., 2017). Instead, only morphological abnormalities are observed in these mice, as they display increased width at pericyte-endothelial junctions (Armulik et al., 2010; Daneman et al., 2010). The effects of deficient pericyte coverage and BBB integrity have also been corroborated in humans using post-mortem brain tissues of AD patients. Analysis of these tissues highlights accelerated pericyte loss in AD tissues when compared to controls, which correlates with the degree of BBB damage and extravascular accumulation of the plasma proteins IgG and fibrin (Halliday et al., 2016). Exposure to cytokines also affects BBB stability as this enhances pericytes migration, a phenomenon observed after rat middle cerebral artery occlusion whereby pericytes detach from blood vessels 1 h post occlusion (Duz et al., 2007). Pericyte detachment may compromise BBB integrity, which in turn causes increased transcytosis of potentially harmful substances such as fibrin and fibrinogen into the CNS, activating microglia and ultimately causing an elevated state of neuroinflammation (Nikolakopoulou et al., 2017; Rustenhoven et al., 2017).

Looking at the multitude of studies regarding pericyte activities during an inflammatory state, it is clear that pericytes play numerous roles in neuroinflammation, making them an attractive therapeutic target to promote neuronal survival by tuning neuroinflammation. Augmenting their anti-inflammatory responses, such as the release of interleukin 33 (IL-33) and trophic factors, may be an avenue worth exploring (Rustenhoven et al., 2017). Nonetheless, more research needs to be completed to devise selective and translational treatments for neuroinflammation.

## Pericytes, Fibrotic Scarring and Axon Regeneration Failure

The fibrotic tissue that develops following CNS trauma and disease is the result of the evolutionarily conserved, ubiquitous, multicellular wound healing process that, under pathological conditions, becomes uncontrolled, thereby causing irreversible fibrosis (Wynn, 2008). On the one hand, the scar tissue allows restoration of tissue continuity. On the other, it acts as a neuronal extrinsic barrier that limits neuroplasticity, axon regeneration and remodeling of neural circuits (Tran et al., 2018). Although described as the absolute barrier to axonal regeneration after SCI, the fibrotic scar has been largely under-researched until recently.

Infiltrating immune cells and stromal cells, like fibroblasts and pericytes, mainly contribute to the formation of the fibrotic scar. Of the different components of the fibrotic scar, pericytes have recently become the subject of intense investigation (Göritz et al., 2011; Fernández-Klett et al., 2013; Birbrair et al., 2014; Dias and Göritz, 2018). However, pericyte identification has often been challenging due to a lack of specific pericyte markers and definitive criteria for pericyte definition (Armulik et al., 2011; Attwell et al., 2016). Using vascular single-cell transcriptomics, a recent study has unraveled a molecular blueprint along the arteriovenous axis. Surprisingly, brain pericytes were not found to form subclusters, thereby suggesting pericytes in the brain are not heterogeneous (Vanlandewijck et al., 2018). Nonetheless, pericyte heterogeneity exists between organs. In fact, lung pericytes do not express CD13 (Vanlandewijck et al., 2018), a commonly used marker for brain pericytes. In light of such an unbiased pericyte classification in the brain vasculature, controversy may arise in the work we discuss below on pericyte heterogeneity and classification in the context of injury and disease.

After tissue damage, pericytes detach from capillaries, extensively proliferate and migrate to the interstitial space where they participate in tissue fibrosis (Göritz et al., 2011; Dias et al., 2018). Stimulated after CNS trauma, such as brain and SCI, macrophages and neutrophils respond quickly to eliminate tissue debris and dead cells (Kono and Rock, 2008). These cells also produce chemokines and cytokines that promote vascularization of the fibrotic scar. By using multiphoton laser ablation of blood vessels followed by time-lapse imaging, a recent study in zebrafish has shown that recruited macrophages extend filopodia and lamellipodia that adhere to both ends of a severed blood vessel and generate pulling forces that help repair cerebrovascular ruptures (Liu et al., 2016). In mice, the number of pericytes associated with sprouting blood vessels increases three to 5 days after SCI (Göritz et al., 2011; Dias et al., 2018). Given that reorganization of the vasculature ensures an adequate supply of oxygen and nutrients to meet the high metabolic demand of regenerating tissues, impaired revascularization may result in delayed formation of the fibrotic scar. Indeed, thymidine kinase/ganciclovir-mediated loss of NG2^+^ cells impairs angiogenesis and completely abolishes fibrotic scar formation after SCI (Hesp et al., 2018; Figure 2). It is important to note that both proliferating pericytes and NG2^+^ glia express NG2. However, pericytes proliferate earlier than NG2^+^ glia following CNS injury. In addition, pericytes and NG2^+^ glia participate in the formation of the fibrotic and glial scar respectively. Whereas lesion sites are enlarged and extended edemas are present 7 days after injury, cell ablation resulted in greater axon regrowth into the lesion 3 weeks after injury (Hesp et al., 2018). When axons are immunolabeled and not traced, however, it is not possible to distinguish between regenerating axons and collateral sprouts from uninjured axons.

Scar formation involves extensive communication between multicellular components (Eming et al., 2014). Therefore, any alteration of one cellular compartment is sufficient to perturb the architecture of the scar that develops after injury. Elimination of proliferating pericytes and NG2^+^ glia impairs the formation of the glial scar as shown by the reduced glial fibrillary acidic protein expression and the presence of discontinuous, loosely entwined astrocytic borders that surround the lesion site 11 days after SCI in mice (Hesp et al., 2018).

Another study has demonstrated that genetic ablation of PDGFRβ^+^ and Glast^+^ (a membrane protein expressed by astrocytes) pericyte, also known as type A, significantly reduces fibrotic scar formation and impairs wound closure after SCI (Göritz et al., 2011), often leaving the lesion site unsealed. Thus, far, it is not known whether the same genetic manipulation may result in the reduction of the astroglial scar. Nonetheless, attenuation of pericyte-derived fibrosis has been recently shown to promote the regeneration of raphespinal and corticospinal tracts after SCI (Dias et al., 2018). Of note, corticospinal axons that regenerate across the site of injury in transgenic mice with attenuated pericyte-derived scarring integrate into functional circuits, effectively improving recovery of sensorimotor function over the course of months after SCI (Dias et al., 2018). The fact that regeneration of the corticospinal tract can be promoted in adulthood by partially eliminating a cellular component of the fibrotic scar is striking, especially considering the lively debate on the extent to which scarring can be beneficial to axon regeneration and functional recovery (Narang and Zheng, 2018).

Pericytes function in a tissue- and context-dependent manner (Holm et al., 2018). In aged skeletal muscle, two types of pericyte subtypes exist. Whereas type-1 (Nestin-GFP^−^/NG2-DsRed^+^) are fibrogenic and participate in scar formation, type-2 (Nestin-GFP^+^/NG2-DsRed^+^) pericytes are myogenic and aid skeletal muscle repair (Birbrair et al., 2013b). Pericyte transplantation has been recently shown to improve skeletal muscle recovery in a mouse model of disuse-mediated muscle atrophy (Munroe et al., 2019). Notably, type-1 pericytes are also present in other tissues including lung, kidney, heart, brain and spinal cord where they proliferate and cluster at the injury site (Birbrair et al., 2014). However, the ability of type-1 pericytes to produce fibrous material such as collagen fibers seems to be organ-dependent (Birbrair et al., 2014), further suggesting pericyte behavior is tightly regulated in a context-dependent manner.

Originally thought to emerge from meningeal cells, the fibrotic scar that develops after contusive SCI more likely originates from collagen 1α1 positive perivascular fibroblasts (Soderblom et al., 2013). Pericytes can differentiate into myofibroblasts and may promote wound contraction, allowing closure of the site of injury. The extent to which collagen 1α1 positive perivascular fibroblasts may be similar to, or originate from, a pericyte lineage awaits confirmation from *in vivo* fate mapping studies. When cultured in a fibrogenic medium, type-1 pericytes originating from skeletal muscle resemble a fibroblast-like morphology (Birbrair et al., 2013a), suggesting that environmental factors like those present at the site of injury may induce certain pericytes to differentiate into fibroblast-like cells. Subretinal fibrosis represents the end stage of age-related neovascular macular degeneration, a risk factor for vision impairment (Lim et al., 2012). Cell lineage tracing following an experimental model of laser-induced photocoagulation in reporter mice expressing collagen 1α1 fused with green fluorescent protein has demonstrated that pericytes associated with choroidal microvasculature infiltrate into the subretinal space between 3 and 7 days after injury, acquire stellate morphology and upregulate expression of fibrogenic molecules, thereby participating in fibrotic scarring (Luo et al., 2018). In light of recent evidence, targeting collagen 1α1 expressing pericytes may represent a valuable therapeutic strategy to preserve vision by suppressing subretinal fibrosis.

Both pericytes and fibroblasts express fibronectin and laminin, permissive substrates for axon growth that also constitute the extracellular matrix of the growth-inhibitory fibrotic scar. Three days after contusive SCI, fibronectin expression increases throughout the lesion site even though fibroblasts are not present in large numbers (Zhu et al., 2015). As fibronectin expression is rather confined around blood vessels at this time, it is likely that pericytes represent the major source of fibronectin early after SCI. By day 7, however, the number of fibroblasts significantly increases and closely matches fibronectin expression (Zhu et al., 2015). Fibronectin transcript is subjected to alternative splicing, generating up to 20 main variants in humans (Kornblihtt et al., 1996). The fibronectin isoform containing the extra domain A has been implicated in pathological fibrosis in experimental models of wound healing (Muro et al., 2003). Genetic elimination of this isoform causes a reduction of fibrotic scarring at chronic vs. acute stages after SCI (Cooper et al., 2018). The elimination also increases axonal density around and within the lesion site, as well as promotes motor recovery after SCI in mice (Cooper et al., 2018). Following contusive SCI, moderate microtubule stabilization through systemic administration of Epothilone B, a drug used for the treatment of cancer, reduces fibrotic scar formation by abrogating polarization and migration of scar-forming fibroblasts (Ruschel et al., 2015). Both laminin deposition and expression of NG2 proteoglycan diminish at the lesion site in rats administered with Epothilone B (Ruschel et al., 2015). Given that pericytes may represent the major source of NG2 expressing cells within the lesion epicenter, it is tempting to speculate that Epothilone B may also affect pericyte behavior after SCI. Indeed, a recent study has demonstrated that Epothilone B administration at 1 and 15 days after complete transection of the thoracic spinal cord in rats causes a reduction in the number of pericytes and extracellular matrix deposition at the injury site (Zhao et al., 2017). The mechanisms underlying changes in pericyte behavior upon Epothilone B administration are not fully understood and deserve attention in future investigation.

Pericytes also express the extracellular matrix protein periostin, a key player in fibrotic scarring. Periostin expression peaks 7 days after SCI, and localizes predominantly in scar-forming pericytes (Yokota et al., 2017). Periostin is thought to promote scar formation by facilitating the recruitment of infiltrating macrophages that, in turn, produce inflammatory cytokines and chemokines that positively regulate pericyte proliferation. Genetic deletion of periostin has been shown to reduce fibrotic scarring and collagen deposition by inhibiting pericyte proliferation through impaired TNF-α signaling (Yokota et al., 2017). Periostin deficiency increases the number of axons within and below the lesion site and promotes some degree of functional recovery over the course of 6 weeks after SCI (Yokota et al., 2017). Moreover, the administration of the periostin neutralizing antibody has proven similarly effective in suppressing the formation of the fibrotic scar as well as promoting recovery after SCI (Yokota et al., 2017).

Thus, accumulating evidence suggests that pericytes play a crucial role in fibrotic scarring and axon regeneration failure. A number of studies demonstrate that a decrease in pericyte proliferation causes a decrease in fibrotic scarring, thus creating more favorable conditions for structural plasticity, axon regeneration as well as recovery of function after CNS trauma and disease. Taking into consideration pericytes heterogeneity and their seemingly contradictory behaviors, a more comprehensive view of the role of pericytes in health and disease may expand their therapeutic landscape.

## Pericytes and Neurodegenerative Diseases

### Diabetic Retinopathy

**Figure 3:**
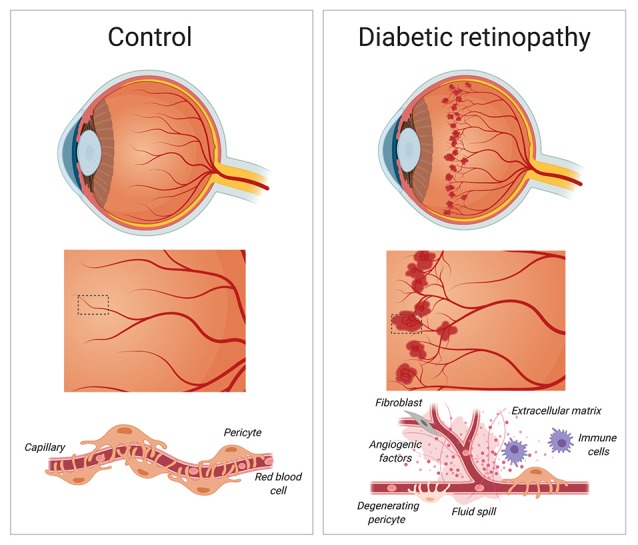
Diabetic retinopathy. During the early stages of diabetic retinopathy, pericytes loss weakens blood capillaries in the retina, causing the formation of microaneurysms, fluid spill and bleeding into the macula. As diabetic retinopathy progresses, the growth of new and irregular blood vessels leads to the formation of a scar tissue that causes blurry vision and loss of eyesight. Created with biorender.com.

Pericytes are involved in a vast network of communication with vascular and non-vascular, parenchymal cells under normal and pathological conditions. Pericytes are associated with non-healing wounds, such as those seen in diabetic retinopathy (Dulmovits and Herman, 2012). Diabetic retinopathy holds a considerable public health burden, affecting nearly one hundred million people worldwide. Although only about one-third of diabetic patients develop proliferative, or sight-threatening diabetic retinopathy (Yau et al., 2012), it is still considered the leading cause of blindness globally in working-aged adults (Armulik et al., 2011). It occurs when damaged blood vessels in the retina cause fluid to leak into the macula, the portion of the eye responsible for clear central vision, ultimately causing blurry vision and loss of eyesight (Heng et al., 2013; Hendrick et al., 2015; Figure 3). Individuals with poor management of their diabetes as well as hypertension are considered at high risk of developing diabetic retinopathy. The course of the disease is irreversible, but there are various treatments used to slow or stop diabetic retinopathy from progressing into later stages such as improving diabetes management through blood sugar control and blood pressure normalization, stopping or slowing the leakage of blood and fluid into the eye using photocoagulation, blood removal through vitrectomy and injecting medication (e.g., anti-VEGF agents) directly into the eye (Hendrick et al., 2015).

The vast majority of the blood in the eye is supplied by the outer blood-retinal barrier, which separates the neural retina from the choroidal vasculature. Endothelial cells, glial cells, and pericytes together comprise the inner blood-retinal barrier (Cunha-Vaz et al., 2011; Park et al., 2017). The retina has the highest pericyte density in the body (Hammes et al., 2002). Not surprisingly, pericyte death has detrimental consequences for retinal function. Mizutani et al. (1996) has demonstrated a relationship between retinal microvascular cell death in human and experimental diabetic retinopathy. Indeed, retinal pericytes nuclei in diabetic patients show fragmentation and pale chromatin staining indicative of ongoing degeneration causing loss of pericytes from the capillary wall (Mizutani et al., 1996). In the absence of adequate pericyte coverage, vascular walls weaken and vessel integrity is lost. Such detrimental structural changes may increase the susceptibility of blood vessels to develop microaneurysms (Wilkinson-Berka et al., 2004).

Numerous studies have attempted to explain the relationship between loss of pericytes and the initiation and progression of the disease. Using transgenic mice with endothelium-restricted ablation of PDGF-B to circumvent prenatal lethality of mice with no pericytes, Enge et al. (2002) have found an inverse correlation between pericyte density and microvascular abnormalities in the retina. Strikingly, a 50% reduction in pericyte coverage sets the stage for the development of proliferative retinopathy (Enge et al., 2002). Of note, similar abnormalities found in experimental models of diabetic retinopathy, such as those used by Enge et al. (2002) are observed in diabetic humans. Therefore, the use of mouse models is crucial to explore basic pathophysiological mechanisms associated with neurodegenerative conditions in the retina. Only by understanding these mechanisms can novel and more effective treatment options be developed.

Hyperglycemia affects individuals with diabetes (Alam et al., 2014). Hyperglycemia is linked to mitochondrial oxidative stress, which in turn causes pericyte death in a mouse model of type 1 diabetes (Price et al., 2012). Bovine and human retinal pericytes cultured in high ambient glucose undergo apoptosis (Beltramo et al., 2004; Suarez et al., 2015). The presence of hyperglycemia in PDGF-B^+/–^ mice (e.g., mice that have lesions similar to those that develop early in a diabetic retina) exacerbates retinopathy with increased density of acellular capillaries and formation of microaneurysms (Hammes et al., 2002). Reduced pericyte coverage also alters angiogenesis in the retina under hypoxic conditions (Hammes et al., 2002). In response to progressive capillary occlusion, the retina responds with either an increase of vascular permeability or with the formation of new immature blood vessels (Campochiaro, 2015). Increasing vascular permeability leads to macular edema, and hypoxic conditions promote excessive neovascularization of the retina (Aiello et al., 1995; Pe’er et al., 1995; Shima et al., 1995; Stone et al., 1995). Members of the VEGF family provide the molecular signals that control vascular permeability and vessel growth. Along this line, expression of the potent angiogenic factor VEGF and its receptor have been shown to increase in diabetic retinas (Penn et al., 2008). Under hypoxic conditions, the half-life of *VEGF* mRNA dramatically increases (Shima et al., 1995). Neutralization of VEGF currently represents the first line of treatment option to prevent excessive neovascularization and macular edema.

Pericyte death in diabetic retinopathy alters the blood-retinal barrier. This tight barrier keeps the eye as an immune-privileged site to protect vision from inflammatory insults. PDGF-B and PDGFRβ signaling actively controls pericyte recruitment to growing retinal vessels (Park et al., 2017). This necessary step is crucial for the formation and maturation of the blood-retinal barrier. Whereas pericyte loss from mature retinal vessels does not cause disruption of the blood-retinal barrier, it triggers the sensitization of endothelial cells to VEGF-A, leading to Forkhead Box O1 (FOXO1)-mediated upregulation of angiopoietin-2 in these cells (Park et al., 2017). This series of steps fuels a positive feedback loop that increases susceptibility to leakage and vascular damage, ultimately ending with a breakdown of the blood-retinal barrier seen in diabetic retinopathy (Park et al., 2017). In mice, genetic ablation of angiopoietin-2 as well as administration of a blocking antibody directed against angiopoietin-2 results in the reduction of microaneurysm and vascular leakage (Park et al., 2017).

Current treatment options for diabetic retinopathies are invasive, have limited effectiveness and mostly address the chronic stages of the disease with minimal improvement in repairing vision (Heng et al., 2013; Hendrick et al., 2015). Management of glucose, blood pressure, and lipid levels along with regular checkups represent the best way to prevent diabetic retinopathy. Even with early diagnostic methods in place, many of those with diabetes will still develop diabetic retinopathy. A direct mechanism has yet to be found on how diabetes causes pericyte death in the retina, but chronic hyperglycemia is currently a key suspect (Hammes et al., 2002).

### Alzheimer’s Disease

**Figure 4:**
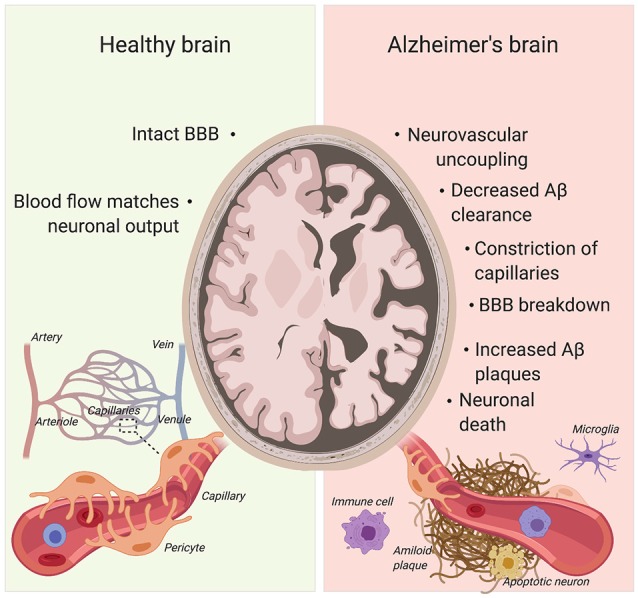
Pericytes’ role in Alzheimer’s disease (AD) pathogenesis. Pericyte degeneration causes neurovascular uncoupling and blood-brain barrier (BBB) breakdown. Through the accumulation of blood products, BBB disruption triggers vascular-mediated secondary neuronal injury, degeneration and cognitive impairment in AD. Given that pericytes are able to clear Aβ, pericyte loss also results in Aβ accumulation in the brain. Aβ can cause constriction of brain capillaries at pericyte locations. Created with biorender.com.

AD already affects 11% of people aged 65 and older and 32% of people aged 85 and over (Alzheimer’s Association, 2015). Due to our aging society, this figure is only set to increase with the incidence of AD expected to double by 2050 (Hebert et al., 2001; Alzheimer’s Association, 2015). AD comes with a plethora of pathophysiological abnormalities such as BBB breakdown, microvasculature irregularities and neurodegeneration (Figure 4; De Strooper and Karran, 2016; Kisler et al., 2017a).

BBB breakdown is present in many neurological diseases, such as motor neuron diseases, multiple sclerosis, stroke and AD (Rustenhoven et al., 2017). The cognitive deficits that AD patients experience have been recently attributed to the breakdown in the BBB (Nation et al., 2019). Numerous studies researching BBB breakdown in AD have discovered a link between pericyte density and BBB integrity (Sengillo et al., 2013; Halliday et al., 2016; Nation et al., 2019). In the brain, pericytes and vascular smooth muscle cells represent the major cellular source expressing PDGFRβ. In individuals with mild cognitive impairment, as well as in PDGFRβ^+/–^ mice, soluble PDGFRβ (sPDGFRβ) increases in the cerebrospinal fluid (CSF) following pericyte loss (Montagne et al., 2015), suggesting sPDGFRβ may serve as a biomarker for pericyte death. Interestingly, human brain pericytes shed sPDGFRβ during cell injury such as hypoxia (Sagare et al., 2015). A significant increase in CSF sPDGFRβ is observed in AD patients with a more advanced clinical dementia rating (Nation et al., 2019). sPDGFRβ remains a reliable indicator of cognitive dysfunction even when controlling for Amyloid-β (Aβ) and Tau deposits (Nation et al., 2019), the two classical markers of AD (Ittner and Götz, 2011). Another study has found a negative correlation between BBB leakage and neurological scores (mini-mental state examination). AD patients and individuals with mild cognitive impairments had increased BBB leakage compared to healthy controls (Skoog et al., 1998; van de Haar et al., 2016). These results display the importance of a fully functioning BBB and the therapeutic potential of increasing pericyte viability for the treatment of mild cognitive impairments. The scores from the clinical dementia rating can report very mild (0.5) and mild (1) cognitive impairments and sPDGFRβ is the only consistent predictor of progression through the scoring, as Aβ_42_ differences can only be found in scores between 0.5 and 1 (Nation et al., 2019), suggesting that pericyte coverage may be used as early biomarker when assessing cognitive deficits.

Pericytes play a role in regulating cerebral blood flow by acting on capillaries (Peppiatt et al., 2006). In order to function properly, the CNS requires the cerebral blood flow to match the neuronal output, also known as neurovascular coupling (Kisler et al., 2017b). Throughout the release of messengers including prostaglandin E2, neuronal activity and the neurotransmitter glutamate can dilate capillaries by relaxing pericytes (Hall et al., 2014). In contrast, buffering astrocyte Ca^2+^ effectively inhibits neuron evoked capillary dilation (Mishra et al., 2016). Although the research does show overwhelming evidence for pericyte’s contribution to cerebral blood flow, as with anything relating to pericytes, the heterogeneity of these cells can provide issues. The differing morphology and protein expression can hint towards a variety of functions; for example, pericytes closer to the arteriole end of the capillary bed express more α-smooth muscle actin and have more circumferential processes, suggesting a more contractile role than pericytes that are in the middle of the capillary bed and express less α-smooth muscle actin (Attwell et al., 2016). However, through extensive animal studies, a strong relationship between pericytes and cerebral blood flow has been identified, if not free from all doubt. PDGFRβ^+/–^ mice display a moderate loss of pericyte coverage of the brain capillary wall (Bell et al., 2010). Using these mice, a recent study has addressed the extent to which pericyte degeneration affects neurovascular coupling. Interestingly, PDGFRβ^+/–^ mice show a global reduction in cerebral blood flow response to neuronal stimuli, leading to neurovascular uncoupling (Kisler et al., 2017b). Focal changes at the capillary level are also observed, as PDGFRβ^+/–^ mice take significantly longer to reach 50% dilation compared to control littermates (Kisler et al., 2017b). This phenomenon is not seen in the arterioles where pericytes are not present, suggesting that the decrease in capillary dilation time and reduction of global cerebral blood flow is a consequence of pericyte deficiency. AD and other dementia patients frequently show focal changes in brain microcirculation which have been associated with cognitive impairments; PDGFRβ^+/–^ mice have significantly reduced capillary length which also coincides with an age-dependent decrease in memory and learning tasks (Bell et al., 2010; Leeuwis et al., 2017). As pericyte death reliably indicates very mild cognitive impairments (Nation et al., 2019), it is likely that pericyte death is not only a symptom of AD but may also be a potential cause of AD. Hypoxia influences amyloid precursor protein (APP) mechanisms that lead to an increased production of β- and γ-secretase and therefore increased Aβ production (Zlokovic, 2011). This suggests that pericyte loss may be an early event in the progression of AD, as pericyte loss reduces cerebral blood flow, thus creating potential hypoxic areas throughout the brain. Although young PDGFRβ^+/–^ mice do not display any difference in neuronal excitability compared to control littermates, older mutants (6–8 months old) with progressive pericyte loss show abnormalities in cortical depolarization, peak amplitude and response latency (Kisler et al., 2017b). Furthermore, these mice also exhibit a decrease in neuron density in the S1 cortex and CA1 hippocampal region and changes in burrowing and nest-building behavior that are considered early signs of AD in mice (Deacon, 2012; Kisler et al., 2017b). Using a genetic mouse model to directly ablate pericytes with diphtheria toxin, Nikolakopoulou et al. (2019) recently showed that loss of pericytes caused BBB breakdown, loss of blood flow and neuronal death with consequent behavioral deficits in 2–3-months-old mice. Ablating pericytes reduced the levels of the neurotrophic growth factor pleiotrophin. When pleiotrophin was restored through CSF infusions, the neuronal loss and behavioral deficits observed were prevented (Nikolakopoulou et al., 2019), highlighting a potential neuroprotective element that pericytes may possess. This is further evidence of the entrenched relationship between the vasculature and nervous system and opens a possible avenue for research regarding neurodegenerative illnesses and pericytes depletion.

Another study found that Aβ causes constriction of brain capillaries at pericyte locations in both human brains as well as in the somatosensory cortex of a transgenic mouse model of AD. Specifically, the application of soluble Aβ_1–42_ oligomers to human brain slices is sufficient to evoke capillaries constriction (Nortley et al., 2019). Capillary constriction is mediated through NADPH oxidase 4 (NOX4)-mediated production of reactive oxygen species in pericytes (Nortley et al., 2019). Further analysis suggests that reactive oxygen species evoke constriction through activation of the endothelin-1 receptor (Nortley et al., 2019).

Together, recent evidence demonstrates a strong association between the neurodegeneration that AD patients experience and a disrupted microvasculature, thereby corroborating an old hypothesis about potential causes of AD (Kimura et al., 1991; de la Torre and Mussivand, 1993; Thomas et al., 1996).

Additional evidence for pericyte importance in protecting from AD lays in common genes that have been identified as risk factors for AD. Apolipoprotein E4 (APOE4) is associated with late-onset AD and carriers of the gene have significantly less pericyte coverage compared to APOE3/2 carriers (Halliday et al., 2016). Presenilin 1/2 (PSEN1/2) are the most frequent genes associated with autosomal dominant AD and carriers of these genes have less *PDGFRβ* mRNA and less PDGF-BB binding sites (Sweeney et al., 2016). Thus, it is no coincidence that genes that are most commonly associated with various types of AD impact pericyte density and survival.

To now, we have only discussed indirect roles that loss of pericytes and capillary constriction can have in the pathogenesis of AD. Pericytes also have direct actions in clearing Aβ *via* the low-density lipoprotein receptor-related protein 1 (LRP1) protein (Sagare et al., 2013; Sweeney et al., 2016; Ma et al., 2018). When intracellular Aβ accumulation overrides the clearance capacity of pericytes, it causes a loss of pericytes that results in an increase in Aβ preventing mesenchymal stem cells from transitioning into pericytes (Xu et al., 2018). In this instance, pericytes offer an attractive possibility as a therapeutic target. If a drug is devised that could target pericytes and prevent cell death or increase the number of mesenchymal stem cells transitioning to pericytes, a greater amount of Aβ protein may be cleared, hence slowing down neurodegeneration in AD. The potential of targeting pericytes or mesenchymal stem cells as a treatment option is evidenced in a recent study where mesenchymal stem cells are differentiated into pericytes and stereotaxically injected into the brains of 18–20 months old APP/PS1 mice (Tachibana et al., 2018), a mouse model of AD. Under such experimental conditions, transplantation of differentiated pericytes results in an increase in the microcirculation in the ipsilateral compared to the contralateral side and reduces insoluble levels of Aβ_40/42_ (Tachibana et al., 2018). When LRP1 is knocked down in pericytes, the reduction in insoluble Aβ_40/42_ is substantially less, but a decrease is still observed suggesting that pericytes may use multiple methods of clearing Aβ (Tachibana et al., 2018).

All this information taken together paints pericytes as an integral cell type in the pathogenesis and progression of AD. Due to the multitude of effects that pericytes have in different functional areas of the brain such as the BBB, cerebral blood flow and clearing Aβ, they are becoming an attractive target for potential treatments of AD.

## Conclusions

After being neglected for many years, pericytes have recently become the focus of an emerging field of research indicating that pericyte dysfunction plays a role in the onset and progression of neurodegenerative diseases as well as tissue fibrosis and lack of motor recovery after SCI (Mizutani et al., 1996 ; Göritz et al., 2011; Li et al., 2017; Nation et al., 2019; Nortley et al., 2019). Despite the development of new genetic tools and cutting-edge imaging techniques that allow labeling and visualization of pericytes “at play” (Proebstl et al., 2012), studying pericyte behaviors remains complicated due to lack of specific markers and definitive criteria for pericyte definition. Ontogeny, localization, morphology and gene signature are all variables that contribute to a variety of complex pericyte behaviors (Vanlandewijck et al., 2018). To date, the extent to which these variables influence pericyte behavior following CNS injury and disease remains fragmentary (Sidebar A). In response to CNS injury and inflammatory mediators, pericytes can constrain blood capillaries (Hall et al., 2014; Li et al., 2017), participate in immune responses (Stark et al., 2013) and contribute to BBB disruption and tissue fibrosis (Göritz et al., 2011; Hesp et al., 2018). Whereas these conditions may not foster neurological recovery, pericytes also play a crucial role in blood vessel formation and maintenance. Vascular normalization of the injury site may be necessary to improve blood flow and perfusion, not only allowing the diffusion of oxygen and nutrients but also the elimination of toxic waste materials to support the functional reorganization of neural networks following injury and disease. Vascular bridging across the lesion site may also provide a permissive substrate to enhance axonal growth and regeneration. As pericytes are plastic and motile during vascular development, they may also serve as carriers for the delivery of therapeutic agents directly at the injury site. In the presence of appropriate stimuli (Gaceb et al., 2018b), pericytes can secrete trophic factors beneficial to repair and restore function to injured neurons. In turn, we hope to encourage a more comprehensive view of the role of pericytes and stimulate work aimed at understanding how to modulate pericyte behavior either alone or in combination with other approaches for therapeutic gain.

SIDEBAR 1 **In Need of Answers**

1.  How many different classes of pericytes really exist through the CNS?

2.  What are the gene signatures of injury and disease-associated pericyte populations in the brain and spinal cord?

3.  How is pericyte behavior controlled in a context-dependent manner?

4.  Provided with the appropriate stimuli, do pericytes promote axon regeneration and CNS repair following injury and disease?

## Author Contributions

FL, JP and AT: conceptualization, visualization and writing. AT: supervision, review and editing.

## Conflict of Interest

The authors declare that the research was conducted in the absence of any commercial or financial relationships that could be construed as a potential conflict of interest.
